# Operative Dentistry

**Published:** 1884-05

**Authors:** J. M. Hurtt

**Affiliations:** Peoria, Illinois


### Operative Dentistry.

BY DR. J. M. HURTT, PEORIA, ILLINOIS.

Read before the Illinois Dental Society.

This subject commands an attention to-day of such importance as to require at the hands of prominent dentists the exercise of keen perceptions and calm, steady steps, that the younger men be not led astray. As to whether the doctors of Dental Surgery are to remain such, or are to become doctors of medicine as well as of dental surgery, the events of the past few years are molding an answer. The Missouri Dental College has, for many years, held out as an inducement to her students the advantage of becoming M. D's. as well as D. D. S's. A great advance was considered as having been made when Harvard University added a dental chair—it was a wise act following professional advancement. Step by step the movement has progressed, until Michigan and other universities have added full dental colleges. It was left for Illinois to carry out the idea of a Dental Infirmary—not only for the education of dentists, but to prepare physicians to understand more thoroughly the needs of the dental profession at their hands than could be taught in medical colleges by simply adding a chair in dental surgery.

Many dentists find it difficult to reconcile themselves to the position taken by the American Dental Association that "Dentistry is a part of Medicine." The correctness of the position of the Association becomes apparent on examination.

Medicine is defined as "The healing art; a science, the object of which is the cure of disease and the preservation of health. Occasionally it is used to comprehend all the branches of the healing art; at others to comprise one great division, in contradistinction to *surgery* and *obstetrics*."

Surgery it defined as, The part of the healing art which relates to external diseases: their treatment; and, especially to the manual operations for their cure. Also a surgeon's office.

"That is an exceedingly crude and erroneous conception which regards surgery as a mere handicraft, or as an art restricted to the treatment of external disease. It will never be possible to draw the lines which separate this department of scientific knowledge from that of medicine; the two are indissolubly one, and all attempts to divorce them detract from both the dignity and power of the surgeon." (*Agnew's Surgery*—Introduction.)

The dental surgeon is, literally, one who operates by the hand on the teeth. But he is defined as a "dentist, or one who devotes himself to the study of the diseases of the teeth, and their treatment." So that the dentist is a surgeon, strictly; but where shall the line be drawn if he would be progressive, for he cannot stop here? The diseases with which he meets defy the beautified excavator, lovely foil, and exquisite mallet—they defy even the oxychlorides, oxyphosphates, gutta percha, quill, paper, horn, asbestos, and cavity cap. These may be applied by the gentlest touch of the gifted surgeon; but a something beyond is required not indicated in these—a something that may be employed with or before them, which the hand cannot reach. The suggestion indicates knowledge, and that implies medical information. "The treatment of dental caries is both medicinal and operative." (*Oral Surgery*—Garretson--2nd ed., p. 273.)

To illustrate:--A dentist without any education in medicine is applied to for his services. He finds a tooth decayed, but neither pulp, exposure, nor soreness. He proceeds to fill the cavity. This he may do from a purely mechanical standpoint, barring his information in dental anatomy, providing his patient is able and willing to submit to his painful procedure; or the tooth is not excessively sensitive—otherwise he would find medical information to be a valuable acquisition. In a day or two the patient returns complaining of toothache in that and the adjacent teeth-- the one filled is too long, so that the others cannot be closed with comfort. The dentist sees nothing wrong with the tooth he filled. "It is sore, but the nerve was not exposed, and it didn't hurt while filling"--he is without a remedy, and recommends extraction.

On the other hand, the dentist who has a medical education, having committed the same error in diagnosis, at once sees his mistake in not discovering a pulpless organ--proceeds to apply medicine to relieve the congestion, dresses down the offending organ as soon as the operation is bearable, or the antagonist if allowable, and opens the pulp cavity, and prescribes a treatment, local or general--either, both, or possibly neither, as may be indicated--and thus saves the tooth.

From this standpoint, Operative Dentistry is that department of medicine which is concerned in the hygienic preservation of the teeth, and the treatment of their diseases by medicines, minerals, and metals. It is the application of dental science. Dental operators are interested in the anatomy and histology of the dental tissues, etiology of dental diseases, and the special history of dental caries. The study of this department invites investigation, from prenatal influence to the last stage of disease necessitating extraction. The position implied is that of dental surgery. This demands for intelligent action, an acquaintance with the body in health and in disease, the relation of each to the other, and the cure of disease. This will enable the operator, with the least loss of the tissues, to perform an operation making the organ useful.

Since the first investigation of this subject, it has been a source of considerable interest to observe the different positions occupied by men who advanced the theory that the dentist was almost purely a mechanic. Not that to be a mechanic is, of itself, degrading, but that he labors to produce angles, curves, and straight lines, in or out of harmony with his idea, if he has any, of the required physiognomy. The surgeon, on the other hand, operates to produce, without regard simply to mechanical lines, but in imitation of nature, such a conservation of the tissues, as will effect healthfulness and harmony of design, which will secure the most useful restoration from the diseased conditions.

In an examination of the teeth with a view to operating, the dental surgeon with a medical education ought to ascertain the hereditary predispositions of the patient, the condition of the mouth and of the teeth, and habits of life. He should make careful deductions from these and what he sees and hears at the first or second sitting. From this he learns as by intuition, whether he can attempt a reformation of the habits of life, with a view to improving both the general system and the teeth. He seeks a conservation of the teeth by artificial restoration, or displacement, the healing of the remaining portion of decayed teeth, and health for the others. To this end the medical, surgical, and dental sciences and arts are made servants. If the manipulative ability is in companionship with the indicated qualifications, the best of dental services is rendered.

On the other hand, if the mechanical dentist should obtain this general information, to what purpose could he use it, as he has no general data for its special use. He therefore must operate only with a view of accomplishing the mechanical without the physiological restoration. Let not this position be misunderstood, for should this information come under proper training at the hands of experienced educators, and he shows proficiency with approved data, then he becomes a dental surgeon—his mechanical abilities will be great gain.

With the specialist of medicine the desire to promote health should overshadow all other considerations. If the patient is not in a condition to be operated on, to operate would endanger the advantages to accrue from it, so that preparatory steps are necessarily taken to gain the object of the visit. In the examination of a cavity there may be found a fungus growth of the gum to deal with—hypersensitiveness of the dental tissue with soft dentine; the pulp may be near; and, as there often is, an insufficient supply of nutrition. Any of these may be accompanied by an abnormal aversion to the operation. In female patients this dislike is often an unavoidable concomitant of gestation, or the catamenia. In male patients the reluctance may come from dissipation, nuerasthenia, or muscular debility. While in both the aversion may be from protracted illness, or the result of grieving over misfortunes. Each of these conditions requires careful management, and each of the disorders special treatment, governed by a consideration of the most prominent affection.

The diagnosis, prognosis, and treatment of many of the diseases of the jaws, of the teeth, and of those caused by sympathy, require a management found only in connection with a general course of medicine.

While the physician should be qualified to treat the general systemic diseases, so should the dentist be to treat the special diseases of the teeth. If a person demands relief from a tooth which is so sensitive that the touch of the lips or of the tongue gives intense pain, to heed a command to extract would not be right, and opening the pulp cavity unbearable, or useless if the root had been filled reliably. A resort to medical treatment must therefore, at first, be the only remedy. Often systemic treatment is the only means of relief. The extreme sensibility to the touch may arise from either of several causes—the immediate cause being pericementitis or periostitis. The exciting or primary cause may be a dying or putrefying pulp; inflammation of that organ from thermal changes; mechanical force from within a cavity of decay; or as the result of a protracted operation. A too forcible occlusion by an antagonist may be the cause, either in the malposition of the tooth, or from striking a filling that had been left too full, the expansion of the amalgam, or from the thickened membranes forcing the tooth up in its socket. The remote cause often is excesses in business or habits; a cold, fever, or a sympathetic relationship with some other diseased organ; or a general dyscrasia. From whatever cause, medical treatment, either local or general, or both, may be indicated. In similar cases, will any dentist assume that the mechanical dentist, without a medical education, is as well qualified to administer relief, with the retention of the organs, as the dentist who has the additional qualification of a medical education? Extracting the offending tooth may or may not relieve. A mechanical dentist, or one without the training and information obtained by a medical education may remove the wrong tooth. His patient may indicate incorrectly, as they often do, a tooth as offending, when the pain is due to reflex nervous action. This error will not often occur at the hands of a dentist having a medical education. He will generally investigate; and having recourse to and confidence in medicine, will seek relief through treatment by hygiene; by local applications, and, if necessary, by administration, giving ample time for recovery, and then, by the mechanical means at his command, prepare the tooth through its roots, to break up the local disease. When the patient has sufficiently recovered for the necessary operation, he will restore the organ to usefulness.

The more that is known of dentistry and dentists, the greater is the surprise to hear men decry a medical education for the dentist. These men are honest, and honorably desire the professional good; but either they are wrong, the definition of medicine as applied to physicians and the human body in health and in disease is incorrect, or dentistry is a part of medicine. The definition is not disputed, nor the proof shown that dentistry is a distinct profession, having no connection with medicine. On the contrary, the investigation incited has led to a broader and more thorough qualification in medicine than before, and the profession of dentistry is already, in many quarters, at the threshold of yielding up its identity as such to the greater. Another positive mark in association with the M. D., is the action always taken in his favor. From the common people of a neighborhood, in a village, to the highest organized body of the dental fraternity—one and all—hail him as the superior man in qualification when compared to the D. D. S., or dentist. In addition to the qualification for the treatment of general diseases, there are local disorders which demand the physician's attention, and require careful judgment in local applications, as well as in general treatment. To qualify for these to the neglect of general disease would be to lose his object. There are no local diseases to which he ought to turn attention that affect the system more than those of the oral cavity. While he cannot treat them in detail, he should know their etiology, symptomatology, and differential diagnosis. He is then less liable to misdirection in the diagnosis on which this knowledge often depends.

As evidences of the necessity of a medical education for dentists and a dental education for physicians, it is desired to examine a few of the phases of a common office practice; and it may be proper to begin with the treatment of some diseases incident to the deciduous teeth.

Sometimes in the treatment of hidden diseases in children's teeth, the utmost care is necessary to determine the exact location and cause of pain. A child who had been under the administration of quinine and iron for intermittent fever and mumps, by mistaken diagnosis, was kept from sleep for seventy-two hours. It was constantly placing the finger in the mouth over the posterior teeth, as if to indicate the locality of the pain. Investigation developed the fact that the child had fallen a year previously, striking its mouth against the stove plate, which loosened the upper front teeth. This led to the belief that death of the pulp of these teeth had taken place as the result of the fall, and was indicated by the child in placing its fingers on the posterior teeth to avoid the anterior. Reasoning that reflex nervous action would cause healthy teeth to act from sympathy, and that the front teeth were too sore to touch, and none of the back ones decayed, the child indicated the posterior teeth, and avoided the front ones. The parotiditis was also explained as the result of sympathy. Therefore, the diagnosis was—pericemental inflammation. A mixture of aconite and iodine was applied to the gums over the anterior teeth. The next morning there was less pain, but the gums under the lip were swollen. The teeth were extracted. The pain ceased, and all traces of the trouble disappeared in two days without further treatment.

Two deciduous incisors were forced from the sockets by an accident, and were replaced, after applying carbolic acid and glycerine to the sockets, and gave no further trouble.

A physician who sent a child to a dentist for the treatment of a first permanent molar, was afterwards called to see the child at its home, suffering pain in the same tooth. He tried to relieve it, but failed. Having informed the parents that it was only a baby tooth, he extracted it. In his medical education, had he been shown the necessity of an acquaintance with dentistry, he would have known a first permanent molar. Had the dentist been qualified in medicine *he* would have been called, and could have saved the valuable member.

There are few operations performed on the teeth that do not require the use of medicines, either within or about them. In replanting a tooth for a medical student both the patient and his preceptor desired to try systemic treatment to relieve the supervening inflammation. Three days of administered treatment by the physician, gave no relief, and but one application by the dentist restored the organ to permanent usefulness. It cannot be said that the previous treatment had nothing to do with the happy result. Hypersensitiveness of an upper molar by the permanent removal of a tobacco quid from its accustomed place, was relieved by iodine, although several dentists declared extraction absolutely necessary. Carbolic acid crystals heated very warm will relieve violent toothache; and applied to the gum over an abscess, and the lancet moistened with it, a painless operation can be slowly performed. Administered arnica will relieve traumatic toothache. Jamaica dogwood extract, applied, will relieve pericementitis after filling a tooth.

Lacto-phosphate of lime syrup, given in dessert-spoonful doses twice a day, relieved morning sickness in gestation during three periods. The teeth were not acted on as is usual when the mother is frail and the teeth susceptible to such action. The walls around the fillings, which were of gold, remained unharmed, and only one new filling was needed during the entire time.

Arriving at home from a journey, a dentist found a young man suffering with pericementitis by pressure of gas from a putrescent pulp. The offending tooth was not decayed, but so sore to the gentlest touch, and even to slowly continued force, as to make an operation to relieve the distress unbearable. Tincture of plantago major was administered—twenty drops to half a glass of water—to be taken in dessert-spoonful doses every hour. Next morning the soreness had disappeared, the tooth was opened and proceeded with, as is usual in ordinary cases.

The same advice, often repeated, will be necessary to most mothers about their own and their children's teeth. Especial care should be taken to guard against the pernicious habit of eating between meals. Bread, meat, fruit, sugar, are injurious when eaten between meals. Close observation will show that decay is accelerated, and generally hastened, by this habit.

Many cases of persistent pericementitis, in even the advanced stages, have been cured by the application of ice-water compresses. Teeth having such excessive sensibility as to preclude the possibility of an operation at the first sitting may become so far advanced towards restoration, by the application of the compress, as to allow an operation at the next sitting. In all pericemental diseases, the occluding or occluded tooth, or both, must be ground down, when possible, until they do not touch, to effect a speedy restoration.

Seldom is a cavity excavated without the application of some obtunding agent to relieve sensibility from either the touch or thermal changes. A denuded root; sensibility from chemical or mechanical abrasion; pulp exposure, and every variety of toothache, receive attention from a medical standpoint. Even condemned old roots, and those with worthless crowns, become firm and invaluable for the reception of the beautiful porcelain crowns. The paper and its discussion on "Dioxide of Hydrogen" in the treatment of blind abscesses, before the American Dental Association last year, ought to be in the hands of every member of this Society.

From Dr. C. A. Harris down to the present time, is it not a fact that most of the professional vigor and efficiency which have urged the profession forward, and made the way clearer, more easily traveled, and placed dentistry among the most honorable pursuits, been occasioned by association with and from the influence of men who were either students in medicine or were physicians? It is admitted that capable operators in the line of mechanical abilities have executed fine fillings, elaborated, and simplified processes for attaching artificial crowns to crownless roots; but let me ask, what prevented the calamity that threatened these exquisite fillings from supervening troubles—more difficult to control than the toothache from the previous decay of the tooth? And, also, from whence the information by which the preparation of the roots were made reliable for the reception of those crowns, and what suggested their possible utility, if not the study and application of medicine?

How came the reception and honorable mention of American dentists at London, but the advance in the principles of medicine?

The profession must be largely benefited in her general status by the membership in, and association with, the American Medical Society. This ought to prove the most stable position occupied by the profession, not excepting those advances made by the addition of chairs in dentistry in universities and medical colleges.

From "Harris' Principles and Practice of Dental Surgery" to "Coleman's Dental Surgery and Pathology," the underlying principle governing all the productions were emanations from the "theory and practice of medicine" as applied to dentistry. Even the higher dental mechanics hinge on the same applied principles. White's and Stocken's Materia Medica are growths of professional necessities from the practice of medicine in dentistry. To read these materia medicas and look up the medicine of dentistry is to learn whither the profession is drifting, as well as how little is known of these things, and how much more can be practiced in the average dental office.

Those dentists who oppose, as unnecessary, the acquisition of the degree M. D., by the dental student as a qualification in dentistry, are honest in their convictions, and believe that the dental colleges ought to present in connection with operative dentistry such a medical education as will be sufficient for all the dentist's needs. The Chicago dentists, by their dental infirmary, strike the middle ground, and add another step in the direction of a satisfactory solution of this puzzling and far-reaching question. It is certainly not the medical information, but the ability to utilize the acquisitions of medicine that makes an M. D., D. D. S. a better dentist than a D. D. S. But in any sense the qualification in medicine, together with a special preparation in dentistry, is better than the best preparation in dentistry without medicine. It is to be hoped that no university, dental college, or infirmary will confer on a graduate in medicine a degree in dentistry until the person has proven by actual demonstration that he has qualified; for the best education in medicine, without qualifying in dentistry, will enable no man to creditably practice dentistry. That the title D. D. S. be not underrated, let it be understood that this has been written from a standpoint of great need of medical information in operative dentistry, by one who was advised not to study medicine.

If this seems a digression from the subject of the paper, please remember that medicine is so often necessary in operative dentistry, and the profession seems so slow to see that hundreds of teeth are saved by therapeusis which were formerly consigned to the forceps—it was impossible to let the opportunity slip. The dental student who stops short of a medical education may ever regret this lack in operative dentistry; and the preceptor who advises simply a course in dentistry may not have read the signs of the times. Let not these remarks lower the elevating work of the noble men who have done so well in teaching operative dentistry, for they are to be thanked; but their students, who have gone beyond them and added medicine, are to be twice thanked.

